# Influence of preoperative weight loss on gastric wall thickness—analysis of laparoscopic sleeve gastrectomy histological material

**DOI:** 10.1007/s00423-022-02668-5

**Published:** 2022-09-08

**Authors:** Krzysztof Barski, Artur Binda, Paweł Jaworski, Agnieszka Gonciarska, Emilia Kudlicka, Joanna Żurkowska, Karolina Wawiernia, Marek Tałałaj, Michał Wąsowski, Wiesław Tarnowski

**Affiliations:** 1 Department of General, Oncological and Digestive Tract Surgery, Centre of Postgraduate Medical Education, Orlowski Hospital, Czerniakowska 231, 00-416 Warsaw, Poland; 2 Geriatrics, Internal Medicine and Metabolic Bone Diseases Department, Centre of Postgraduate Medical Education, Orlowski Hospital, Czerniakowska 231, 00-416 Warsaw, Poland

**Keywords:** Gastric wall thickness, Preoperative weight loss, Staple line leak, Sleeve gastrectomy

## Abstract

**Purpose:**

The variables possibly enabling the prediction of gastric wall thickness during laparoscopic sleeve gastrectomy remain undetermined. The aim of the study was to identify preoperative factors affecting gastric wall thickness in patients undergoing laparoscopic sleeve gastrectomy.

**Methods:**

The measurements of the double-wall thickness of gastric specimen excised during sleeve gastrectomy were taken at three locations after 15 s of compression with an applied pressure of 8 g/mm^2^. Statistical calculations were used to determine the influence of preoperative weight loss and other perioperative parameters on gastric wall thickness.

**Results:**

The study involved one hundred patients (78 female; 22 male). The thickest tissue was observed at the antrum with the mean value 2.55 mm (range 1.77–4.0 mm), followed by the midbody, mean 2.13 mm (range 1.34–3.20 mm), and the fundus, mean 1.69 mm (range 0.99–2.69 mm). Positive relationships were found between gastric wall thickness and both preoperative weight loss and age in all three measured locations; *p* < 0.05. In a linear regression model, age and preoperative weight loss were found to be statistically significant and positive predictors of higher gastric wall thickness only at the antrum. Male patients were observed to have thicker gastric wall at all three locations as compared to female patients.

**Conclusion:**

Preoperative weight loss should be considered an important factor influencing gastric wall thickness. Age and gender can also be helpful in predicting the varying tissue thickness. Anatomical region is a key factor determining thickness of the stomach walls.

## Introduction

The global epidemic of obesity is a great burden on world health care [1–3]. Bariatric surgery has been proven to be more effective for treatment of morbid obesity when compared with conservative treatment [4]. Laparoscopic sleeve gastrectomy (LSG) is the most commonly performed primary bariatric procedure worldwide [5]. Despite the beneficial effect on the intended weight loss following LSG, some patients may suffer from major complications, and among them, one of the most unwanted and life-threatening is staple line leak (SLL). If it occurs, mortality risk significantly rises [6]. Pathogenesis of staple line leak may derive from a mismatch between closed staple height and thickness of the tissue being transected. Staples, when closed, need to be formed in an exact B-shape figure to obtain hemostasis and tissue allocation and still to avoid ischemia and gastric wall rupture. Maintaining the balance between tissue creep, stress relaxation, and tensile stress during stapling is fundamental to ensuring staple line integrity [7]. Manufacturers provide stapling reloads with different staple heights intended for use in different tissue thicknesses, which implies an obligation of gastric wall thickness (GWT) awareness and using staples of appropriate height during stapling. The perioperative variables enabling the prediction of gastric wall thickness prior to staple height choice remain undetermined. The relation between patients’ medical characteristics and gastric wall thickness has been investigated, but so far there are no universal conclusions to adopt in clinical practice. A parameter that has not been examined yet in the context of gastric wall thickness is preoperative weight loss (PWL). Currently, available data concerning the beneficial effects of preoperative weight loss on the outcomes of bariatric surgery are inconclusive [8–11]. Nevertheless, we encourage patients to lose their body weight up to 5–10% of the initial body weight before the surgery due to the subjectively greater simplicity of carrying out the surgery. We have observed that in the group of older patients with preoperative weight loss, it was necessary to use reloads with higher staples due to the greater thickness of the transected tissues. To verify these clinical findings, in the present study, we aimed to determine the variability of gastric wall thickness depending on preoperative weight loss and the other perioperative factors based on histological material obtained during LSG.

## Materials and methods

The project of the study was approved by the Bioethics Commission of the Centre of Postgraduate Medical Education (No. 48/PB/2016). The study was launched in 2016. A group of a hundred patients who underwent LSG for morbid obesity was enrolled in the study. Informed consent was obtained from all patients. Criteria of eligibility for surgical treatment were as follows: BMI between 35 and 39.9 kg/m^2^ and obesity-related comorbidities or BMI ≥ 40 kg/m^2^. The exclusion criteria were a history of bariatric surgery and the inability to provide informed consent. All patients were encouraged to reduce their initial body weight by 10% before the surgery. The preoperative management and ambulatory care pathway covered the Polish recommendations for bariatric and metabolic surgery [12]. A detailed database covering patients’ characteristics and wall thickness measurement results was created prospectively.

### Surgical technique

The critical points of each LSG were invariable and proceeded as follows: the stapler appliance started 4 cm from the pylorus; the range of the resection was calibrated using a nasogastric tube (36 Fr); staple line tightness was checked by the methylene blue test; drainage was placed next to the staple line. Endoscopic linear staplers were used for gastric division depending on the surgeon’s preference: Endo GIA™ Universal Stapler with Endo GIA™ Reloads with Tri-Staple™ Technology, 60 mm, Covidien, Medtronic, USA, or Echelon Flex™ Endopath^®^ Stapler with 60 mm Endopath Echeleon™ Reloads, Ethicon Endo-Surgery Inc., Johnson and Johnson, USA. The gastric division was started 4–6 cm proximal to the pylorus. When Echelon Flex™ Endopath^®^ Stapler was applied, one 60 mm green staple cartridge (open/closed staple height 4.1 mm/2.0 mm) was used to transect the antrum, and then blue reloads (open/closed staple height 3.6 mm/1.5 mm) were subsequently applied towards the angle of His. For Covidien Endo GIA™ Stapler, the first staple cartridge used at the antrum was purple (open/closed staple height 3.0, 3.5, 4.0 mm/1.25, 1.5, 1.75 mm) and the division of the midbody and fundus was completed with tan reloads (open/closed staple height 2.0, 2.5, 3.0 mm/0.75, 1.0, 1.25 mm). The detailed data regarding staples’ height were taken from the manufacturers’ manuals.

### Measurements of GWT

**Fig. 1:**
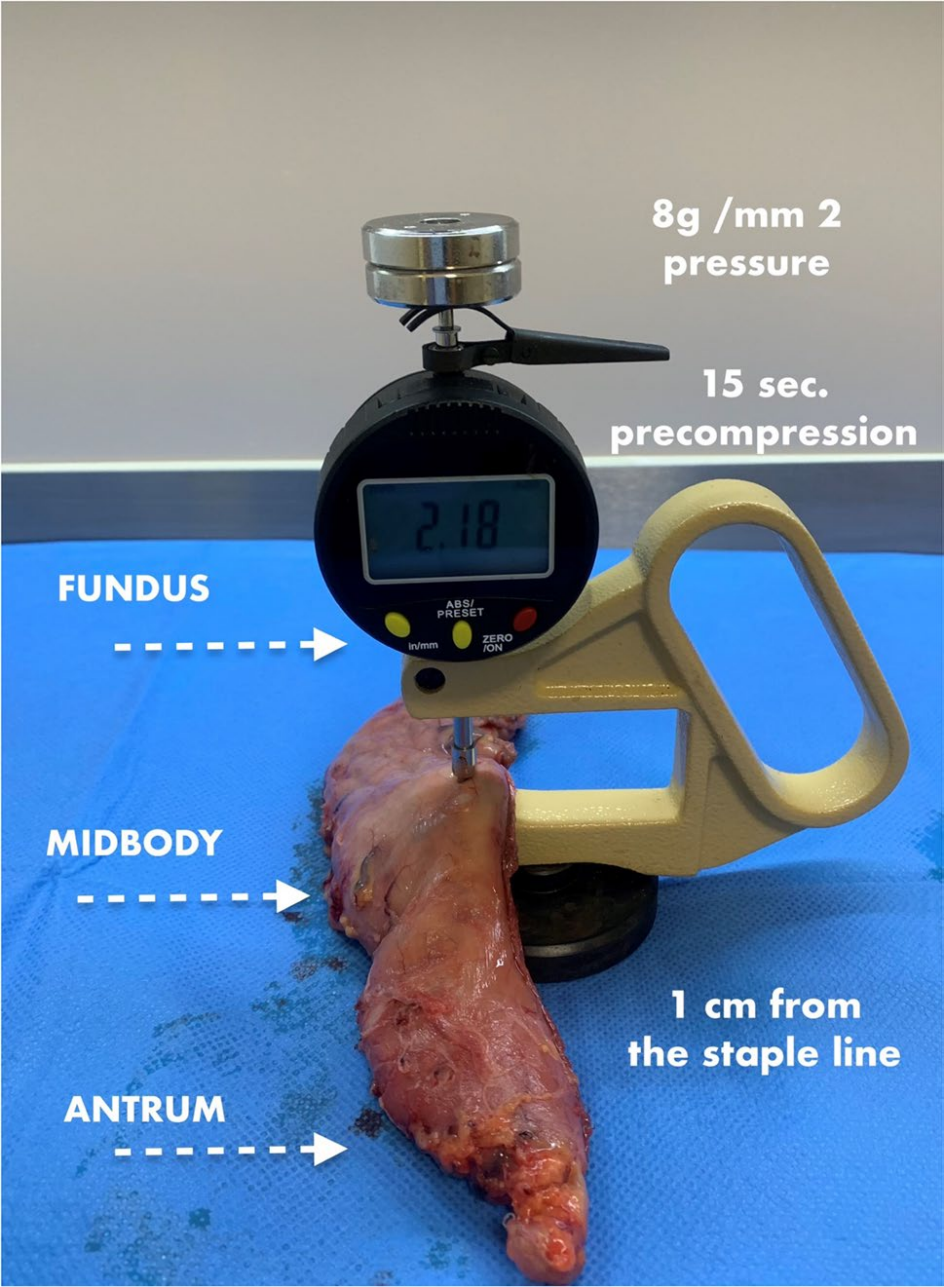
Thickness gauge used in the study

Immediately after the excised gastric specimen was removed from the operative field, the measurements of gastric wall thickness were always taken by the same researcher according to the developed scheme (Fig. 1). The thickness measuring instrument was modified to reach the pressure of 8 g/mm^2^ during the examination, which is, according to the literature, the optimal compression causing no structural tissue damage [7, 13]. The result was recorded after 15 s of gastric wall compression, as it is the optimal duration to reach the proper tissue thickness and not damage it [14]. The measurements of the double-wall thickness were taken at three locations of the excised portion of the stomach: at the antrum, 2 cm up from the distal end of the specimen, in the middle of the body, and at the fundus, 2 cm down from the proximal end. All measurements were taken 1 cm from the staple line.

### Statistical analysis

All necessary calculations were carried out with SPSS Statistics version 25.0 (Armonk, NY, IBM Corp.). The normality of data distribution was estimated with the Shapiro–Wilk test. The analysis of the correlation between BMI, changes in BMI, age, and gastric wall thickness was calculated with the Pearson correlation coefficient. A linear regression model was used to determine whether gastric wall thickness can be predicted based on BMI change and age. The differences in gastric wall thickness at the three measured locations were estimated by the analysis of variance with the Greenhouse-Geiser correction. To reveal which location the gastric wall is the thickest, a pair comparison with the Sidak correction was used. To compare gastric wall thickness at three measured locations among women and men, the independent samples *t*-test was applied. A *p*-value < 0.05 was considered statistically significant for all the analyses.

## Results

The study was conducted on a group of hundred patients (78 female; 22 male) with a mean age of 42 years (range 19–68 years). The mean preoperative BMI loss was 8.1 kg/m^2^, and the mean BMI on the day of surgery was 41.6 kg/m^2^. Maximum preoperative BMI loss expressed as a percentage of initial BMI was 24.6%. Only one patient failed to lose weight before surgery. Patients’ characteristics regarding age and BMI are presented in Table 1. Arterial hypertension and diabetes mellitus/prediabetes were the most common obesity-related comorbidities diagnosed in the study group, respectively, in 45% and 35% of patients preoperatively. The preoperative obesity-related comorbidity rates for other frequent comorbidities were 26% for dyslipidemia and 15% for obstructive sleep apnea syndrome. Detailed data on obesity-related comorbidities before surgery are presented in Table 2.

**Table 1:** Patient characteristics

Variables	*M*	Me	SD	Min	Max
Age (years)	41.8	41.0	11.3	19	68
BMI at the day of surgery (kg/m^2^)	41.6	40.0	5.9	30.9	68.9
Preoperative BMI loss (%)	8.1	7.4	4.5	0.00	24.6

*M*, mean; *Me*, median; *SD*, standard deviation; *Min.*, minimal value; *Max.*, maximal value

**Table 2:** Preoperative obesity-related comorbidity rates

Prevalence of comorbidities in the study group (*n* = 100)	% of patients
Arterial hypertension	45
Diabetes mellitus	19
Prediabetes	16
Dyslipidemia	26
Hypothyroidism	15
Obstructive sleep apnea syndrome	15
Osteoarthritis	12
Reflux disease	12
Gastritis	11
Asthma	10
Gout	4
No comorbidities	18

*n*, number of patients

All procedures were completed laparoscopically with no complications requiring conversion to open surgery. The overall 30-day complication rate was 6%. Early major complications occurred in two patients (2%). Staple line leakage occurred in one patient and was successfully managed with endoscopic gastric stent placement and laparoscopic drainage. One patient developed symptoms of intraperitoneal bleeding on the 1^st^ postoperative day, but no cause was found during relaparoscopy, and the subsequent postoperative course was uncomplicated. There was no peri- or postoperative mortality. The Clavien-Dindo classification was used to grade the complications, and the results are presented in Table 3.

**Table 3:** Thirty-day complication rates (by Clavien-Dindo classification)

Complication	*n* (%)	Clavien-Dindo grade	Treatment
Staple line leak	1 (1)	IIIb	Laparoscopic drainage, endoscopic stent
Splenic infarction	1 (1)	II	Conservative treatment
Staple line bleeding	1 (1)	IIIb	Laparoscopic revision
Nausea and vomiting	2 (2)	I	Prokinetics, proton pump inhibitors
C. difficile infection	1 (1)	II	Pharmacological treatment
Total complication rate	6 (6)		

*n*, number of patients

The thickest tissue was observed at the antrum with a mean value of 2.55 mm (range 1.77–4.0 mm). In the proximal direction, the tissue started to be thinner; the mean thickness of the midbody was 2.13 mm (range 1.34–3.20 mm), and the mean value of GWT at the fundus was 1.69 mm (range 0.99–2.69 mm). The analysis of variance showed statistically significant differences between the performed measurements. Results of gastric wall thickness measurements at the three predetermined locations are shown in Table 4.

**Table 4:** Results of gastric wall thickness measurements

Variables	*M*	Me	SD	Min	Max
Antrum (mm)	2.55	2.52	0.42	1.77	4.00
Midbody (mm)	2.13	2.08	0.34	1.34	3.20
Fundus (mm)	1.69	1.70	0.32	0.99	2.69

*M*, mean; *Me*, median; *SD*, standard deviation; *Min.*, minimal value; *Max.*, maximal value

Statistically significant, positive relationships were found between gastric wall thickness and both preoperative weight loss and age in all three measured locations; *p* < 0.05. It means that as the preoperative weight loss or age increases, the gastric wall thickness also tends to increase, although the degree of correlation for both preoperative weight loss and age was moderate or low for all three locations. No statistically significant correlation was found between the baseline BMI and gastric wall thickness or BMI on the day of surgery and gastric wall thickness in any of the measured locations. Data are presented in Table 5.

**Table 5:** Results of the analysis of correlation between BMI, changes in the BMI, age, and gastric wall thickness in three locations calculated with the Pearson correlation coefficient

Variables		Antrum	Midbody	Fundus
Baseline BMI	Pearson’s *r*	0.18	0.22	0.07
	*p* value	0.066	0.027	0.473
BMI at the day of surgery	Pearson’s *r*	0.05	0.13	− 0.04
	*p* value	0.626	0.192	0.681
PWL	Pearson’s *r*	0.40	0.28	0.35
	*p* value	< *0.001*	*0.005*	< *0.001*
Age	Pearson’s *r*	0.34	0.35	0.35
	*p* value	< *0.001*	< *0.001*	< *0.001*

*BMI*, body mass index; *PWL*, preoperative weight loss; italics: statistically significant

In a linear regression model, age and preoperative weight loss were statistically significant and positive predictors of higher gastric wall thickness at the antrum. No similar relationship was confirmed for either the midbody or the fundus. Based on this analysis, one can assume that if age increases by 1 year, then the thickness of the stomach wall at the antrum increases by 0.01 mm, whereas the loss of initial BMI by 1% increases the gastric wall thickness at the same location by 0.04 mm. A stronger predictor in the created model was the preoperative BMI loss expressed in % (beta = 0.41) compared to the age of the patients (beta = 0.25). Discussed values for antrum are presented with details in Table 6.

**Table 6:** Coefficients of a linear regression model predicting gastric wall thickness at the antrum based on the preoperative weight loss and age

	Coefficients	*β*	SE	Beta	*t*	*p*	Adj-*R*^2^
GWT of the antrum	Constant	1.86	0.14		13.72	< *0.001*	0.28
	Age	0.01	0.003	0.25	2.76	*0.007*	
	PWL	0.04	0.01	0.41	4.56	< *0.001*	

*β*, a non-standardized coefficient; *SE*, standard error; *Beta*, predictors value; *t*, Student’s *t*-test value; *p*, *p*-value; Adj-*R*^2^, adjusted coefficient of the determination; *GWT*, gastric wall thickness; *PWL*, preoperative weight loss; italics: statistically significant

Male patients were observed to have a thicker gastric wall at all three locations than female patients, but only at the antrum and at the midbody these differences were statistically significant (Table 7).

**Table 7:** Gastric tissue thickness—female versus male patients

	Female (*n* = 78)		Male (*n* = 22)		
	*M*	*SD*	*M*	*SD*	*p*
Antrum	2.50	0.43	2.73	0.32	*0.019*
Midbody	2.08	0.34	2.29	0.31	*0.010*
Fundus	1.66	0.33	1.80	0.29	0.067

*M*, mean; *SD*, standard deviation; *p*, *p*-value; italics: statistically significant

## Discussion

The necessity of complex and long-term treatment and life-threatening consequences of staple line leak puts this complication at the top of the list of unwanted events following LSG. The incidence of staple line leak after LSG is estimated to be 2.2% [15]. This paper focuses on gastric wall thickness as a measurable factor that may impact the proper staple height choice used for stapling during the LSG. Properly selecting staples during the surgery is crucial for optimizing the interaction between the transected tissue and stapling device and reducing staple line–related complications. The first analysis of gastric wall thickness from the perspective of bariatric surgery by Elariny gave the basis to create stapler manuals [14]. In subsequent studies, the variability and predictability of this parameter in the context of LSG have been closely investigated and brought the aspect of gastric wall thickness into the discussion about staple line leak [16]. The descending gastric wall thickness from the antrum towards the fundus was confirmed in our study, and it is a significant feature that implicates the staple height adjustment during LSG. Our results correspond to observations made in previous studies using a similar methodology [16]. In the present study, maximal tissue thickness was 4 mm, which meets the upper limit of the size of black reloads designed for extra-thick tissue. Other researchers have observed that the tissue thickness may exceed 4 mm, which raises the question of whether the set of staple reloads should be enlarged [16]. In our opinion, manufacturers should consider extending the range of available cartridges. The scheme of stapler choice should be unified to avoid incoherence of the operative protocols, especially when serious complications such as staple line leak may lead to legal issues.

We observed that patients’ initial and preoperative BMI did not correspond with gastric wall thickness. Similar results were described by van Rutte et al. [17] and Elariny et al. [14]. Rawlins et al. observed that BMI affects the gastric wall thickness only when it exceeds 50 kg/m^2^ [18]. On the surface, for a practicing bariatric surgeon, it may be misleading, but a higher BMI does not necessarily predispose to the use of a cartridge with higher staples.

Preoperative weight loss is advised for patients scheduled for bariatric surgery as it has been observed that it increases excess body weight loss, reduces postoperative complications, and shortens the procedure duration and hospital stay [8, 11, 19, 20]. Although preoperative weight loss may be beneficial for patients undergoing bariatric surgery, usually, preoperative recommendations are difficult to execute in bariatric departments [21]. Based on the inconclusive literature and own studies, some authors do not support the medical necessity of weight loss prior to bariatric surgery [22]. Despite the absence of specific guidelines on this issue, we advise weight loss of 10% of initial body weight as part of the preoperative medical management. Based on our clinical observations of bariatric procedures, we hypothesized that preoperative weight loss is related to a thicker gastric wall. We designed a study to determine whether preoperative weight loss may induce changes in gastric wall thickness and therefore be a hint for the surgeon when choosing a cartridge for the stapler. This hypothesis was confirmed in our study. A correlation report showed a positive relationship between preoperative weight loss and gastric wall thickness at each measured location. Moreover, a designed statistical model revealed that more significant preoperative weight loss is related to the increased gastric wall thickness at the antrum. A positive relationship between older age and thicker gastric tissue in all three measured locations was also found. These observations may be related to changes in the proportion between the muscular and connective tissue, which occurs after weight loss and progresses with age. Further microscopic studies should answer whether there are any specific changes in the histological structure of the stomach after weight loss and whether they are related to age.

An obvious finding of our study, also confirmed in other studies, is the observation of a significant difference in tissue thickness between anatomical regions of the stomach. This observation is the root of the current use of a sequence of stapler cartridges during LSG. A popular explanation of the origin of imperfect staple line formation is inadequate stapler reload choice. So far, there is no official consensus on which stapler reloads should be used during LSG. The sequence of applying cartridges with different staple heights during LSG is not unified. Some surgeons report using only black reloads; some first use green and then turn to gold and blue, while others avoid the black and blue staplers performing LSG. After analyzing the changeability of gastric wall thickness, Huang and Gagner [23] suggested that a good solution would be to develop a device to measure it before stapler firing. When analyzing the influence of gender on the gastric wall thickness, male patients were found to have a thicker gastric wall in all three regions of the stomach, but only in the antrum and the body these results were statistically significant. However, the most recent cohort study by Boeker et al. [24] found that the male gender was associated with thicker tissue in the fundus, which is the most common staple line leak localization.

There were two (2%) early major complications in the study group. Staple line leakage occurred in one patient, and one patient developed symptoms of intraperitoneal bleeding. Nevertheless, based on the results of the present study, we were not able to determine the relationship between the risk of these complications and the height of the staples used. It can be assumed that one of the leading causes of life-threatening complications such as leakage or bleeding may be a mismatch between staple height and gastric tissue thickness [23, 25]. We could draw similar conclusions based on our general experience using staplers during bariatric surgeries. The use of staples in the sequence described in the present study was based on our previous experience in performing LSG. During this period, we recorded only two staple line leaks and three cases of bleeding requiring reoperation out of approximately 1000 LSG performed. If difficulties arose related to the wrong selection of the reloads during surgery, reloads with a greater staple height were adjusted. We were prompted to change both by the results of previously published papers and the initial results of our study. After this modification, we did not notice any staple line leaks, although a few postoperative bleeding complications required reintervention. The suitability of a particular cartridge for each stomach area depends on the tissue thickness at that location. The variability in cartridges’ suitability at the different parts of the stomach underscores the need for a surgeon to be confident of the tissue thickness before choosing a cartridge [23]. A mismatch between staple height and gastric tissue thickness may compromise the integrity of the staple line [23–25]. Manufacturers and most surgeons recommend matching closed staple height to gastric tissue thickness to avoid incomplete staple formation or tissue damage. Contrary to the traditional model of fitting staples to tissue, the results of the study by Abu-Ghanem et al. [26] suggest that the application of tight reloads could have clinical benefits and does not increase the expected rate of leaks. Most surgeons use different staples’ heights based on differences in wall thickness across different areas of the stomach and according to their tactile feeling. The results of the study by Susmallian et al. [27] indicate that the surgeon’s tactile feeling is inaccurate in most cases. Thus a thickness calibration device may help determine the correct staple height [23]. It is difficult to assess whether an increased thickness of the stomach walls we observed in elderly patients and those with more significant preoperative weight loss in the present study would reduce the risk of staple line–related complications. In any case, we cannot draw such conclusions from the present paper. Taking into account the results of both the present study and cited papers, one can assume that the use of reloads with higher staples, e.g., gold/tan or purple, may be beneficial in reducing staple line leaks in case of elderly, male patients and those with higher preoperative weight loss. In our opinion, it is impossible to provide strict and unambiguous recommendations regarding the selection of staples’ heights based solely on the results of this study. Nevertheless, we hope these results may be helpful for bariatric surgeons in their daily practice.

The weakness of the current study may be the lack of assessment of the impact of factors such as type 2 diabetes, arterial hypertension, smoking, age of onset of obesity, years of obesity, and Edmonton obesity staging system score on the thickness of the stomach walls. As is well known, these factors may influence bariatric surgery outcomes and the risk of complications [28, 29]. Some of these factors could also potentially affect the thickness of the stomach walls [26]. On the other hand, in the studies by Susmallian et al. [27] and Boeker et al. [24], no correlation was found between gastric wall thickness and obesity-related comorbidities. It would be worth assessing these factors’ influence on gastric wall thickness in further studies, especially considering that most bariatric patients suffer from obesity-related comorbidities. In our study, various types of obesity-related comorbidities were found in 82% of patients. However, evaluating this topic went beyond the scope of the current study.

The obvious disadvantage of our study is the relatively small sample of patients. The results oblige us to extend the study to a larger patient group to verify the statement about the influence of preoperative weight loss on gastric wall thickness. Based on the results of the present study, we have only confirmed our previous clinical observations that preoperative weight loss increased the thickness of the stomach walls. It can be assumed that the reduction in the stomach volume due to a restrictive diet before the surgery causes an increase in the thickness of its walls. It seems that explaining this phenomenon would require histopathological examinations of excised specimens. These examinations would probably also help assess the mechanisms of the influence of older age and obesity-related comorbidities on the thickness of the stomach walls.

## Conclusions

The anatomical region is a key factor determining the thickness of the stomach walls, which is crucial for staple height matching. Preoperative weight loss, older age, and male gender increase the gastric wall thickness and may be essential factors facilitating the choice of stapler reloads during sleeve gastrectomy. The baseline BMI does not significantly affect the thickness of the stomach walls.
